# Comparative diversity of aquatic plants in three Central European regions

**DOI:** 10.3389/fpls.2025.1536731

**Published:** 2025-03-06

**Authors:** Marek Svitok, Igor Zelnik, Kateřina Bubíková, Mateja Germ, Alenka Gaberščik, Judita Kochjarová, Helena Oťaheľová, Peter Paľove-Balang, Richard Hrivnák

**Affiliations:** ^1^ Faculty of Ecology and Environmental Sciences, Technical University in Zvolen, Zvolen, Slovakia; ^2^ Institute of Botany, Plant Science and Biodiversity Center of Slovak Academy of Sciences, Bratislava, Slovakia; ^3^ Faculty of Forestry and Wood Sciences, Czech University of Life Sciences Prague, Prague, Czechia; ^4^ Biotechnical Faculty, University of Ljubljana, Ljubljana, Slovenia; ^5^ Faculty of Forestry, Technical University in Zvolen, Zvolen, Slovakia; ^6^ Faculty of Natural Sciences, University of Pavol Jozef Šafárik, Košice, Slovakia

**Keywords:** α, β, γ diversity, turnover, nestedness, meta-analysis, ditches, ponds

## Abstract

Freshwaters are among the most threatened ecosystems globally, with biodiversity declining at far greater rates than the biodiversity of the most affected terrestrial ecosystems. There is an urgent need for accurate information on spatial patterns of freshwater biodiversity, a first step in effective conservation planning and management of these ecosystems. We explored patterns of aquatic macrophyte diversity in four waterbody types, rivers, streams, ponds and ditches, across three Central European regions. By analyzing local (α), among-site (β) and regional (γ) diversity, we assessed the roles of these ecosystems as biodiversity hotspots, particularly for red-listed species. Sampling 220 sites across Slovakia and Slovenia, we recorded 113 macrophyte taxa (31% of which were red-listed), with ponds and ditches consistently supporting higher α and γ diversity than running waters. β diversity was primarily driven by species turnover, with ponds displaying high heterogeneity linked to environmental variability. Our findings highlight the conservation value of artificial habitats like ditches and ponds, harbouring significant macrophyte diversity, including unique and threatened species. These results underscore the need to prioritize small waterbodies in biodiversity conservation strategies within agricultural landscapes.

## Introduction

Freshwaters are among the most threatened ecosystems on Earth (Dudgeon et al., 2006). Intensification of land use, eutrophication, flow modification, expanding hydropower exploitation, pollution, changes in biotic interactions due to the introduction of invasive alien species, and climate change are the main direct drivers of freshwater degradation (Sala et al., 2000; Millennium Ecosystem Assessment, 2005; Williams-Subiza and Epele, 2021). The degradation and loss of freshwater habitats occur more rapidly than in other ecosystems, and the adverse effects of these changes on freshwater biodiversity are considerable (Dudgeon et al., 2006; Lacoul and Freedman, 2006; Reid et al., 2019). As a result of all these impacts, freshwater biodiversity is declining at far greater rates than the biodiversity of the most affected terrestrial ecosystems, and this trend is expected to continue (Ricciardi and Rasmussen, 1999). There is an urgent need for accurate information on freshwater biodiversity, as the state of knowledge regarding biodiversity threats is unsatisfactory for many freshwater habitat types (Millennium Ecosystem Assessment, 2005), and inventories of freshwater biodiversity are far from complete in many regions (Dudgeon et al., 2006).

While preserving intact freshwater ecosystems and their biodiversity remains a conservation priority, the call for recognition of the important potential of human-modified habitats to maintain freshwater biodiversity appeared relatively recently (Dudgeon et al., 2006). Regardless of habitat type, identifying biodiversity hotspots is still mandatory for effectively protecting freshwater biodiversity. Early studies in the United Kingdom compared freshwater biodiversity across various waterbodies, from natural to human-modified and even artificial (Williams et al., 2004; Biggs et al., 2007). Davies et al. (2008a, b) expanded the geographic scope by comparing aquatic diversity in agricultural landscapes across Denmark, France, Germany and the United Kingdom. The results of recent studies highlight the role of small waterbodies as hotspots for plant and macroinvertebrate biodiversity (Biggs et al., 2017; Zelnik et al., 2018). In particular, ponds have been shown to support more rare and red-list species than other waterbody types. The high biodiversity values of both natural and artificial ponds have also been demonstrated by other studies (e.g., Boix et al., 2012; Lukács et al., 2013; Bubíková and Hrivnák, 2018a). However, ponds remain a low priority in national and international conservation and environmental legislation in most countries (Hill et al., 2018, 2021).

In addition to ponds, ditches − shallow channels found in agricultural landscapes − can play a key role in preserving biodiversity in human-dominated environments (Svitok et al., 2016; Bubíková and Hrivnák, 2018b). Historic ditches can contribute to cultural heritage and biodiversity conservation (Lin et al., 2020). These habitats are also considered hotspots for macrophyte diversity (Verdonschot et al., 2011; Dorotovičová, 2013; Clarke, 2015). However, other specific habitat type, lakes, can have higher or equal alpha diversity in landscapes with various dominant land use compared to ponds, canals and ditches (Law et al., 2024). Given the importance of spatial processes in community structuring (Wiens et al., 1993), it is unlikely that findings from a limited number of case studies can be easily extrapolated to other geographical and ecological contexts. The diversity of local communities is influenced by the regional species pool, local biotic interactions and abiotic factors, with different environmental variables potentially controlling community structure in different regions. Specifically, freshwater diversity patterns are regionally context-dependent (Heino, 2011). In macrophyte communities, where the significance of spatial processes and environmental factors can vary unpredictably by location, it may be unwise to draw broad conclusions from a few geographical regions (Alahuhta and Heino, 2013; Alahuhta et al., 2017).

In this study, we assessed patterns of macrophyte diversity across a range of aquatic ecosystems in three Central European regions, which primarily differ in altitude and associated climate but share a large majority of species. Compared to studies conducted in Western Europe, these Central European regions were historically shaped by socialist-style agriculture characterized by large-scale monocultural farming and state-directed practices that frequently neglected ecological considerations (Bezák and Mitchley, 2014). Information on the comparative diversity of macrophytes in Central Europe is limited; there is only one study from the Váh River Valley in Slovakia (Bubíková and Hrivnák, 2018b).

The geographic proximity of these regions and the strong dispersal abilities of aquatic plants are advantageous, as they significantly reduce the effect of dispersal limitation on our results. Specifically, we aimed to compare local (α), among-site (β), and regional (γ) diversity of aquatic macrophytes in ponds, ditches, streams, and rivers across Slovakia and Slovenia. More broadly, we tested the generality of conclusions from previous comparative diversity studies within a Central European context. Based on earlier research (e.g., Williams et al., 2004; Davies et al., 2008a; Bubíková and Hrivnák, 2018b), we hypothesize that ponds and ditches serve as hotspots of macrophyte biodiversity in these Central European regions, despite their artificial origin.

To inform conservation planning, we specifically focused on the diversity of red-list species. Aquatic macrophytes are of particular conservation importance, as they include many threatened species (Bilz et al., 2011; Bolpagni et al., 2018) and are relatively easy to identify, making them a valuable proxy group of organisms that are less challenging to distinguish (Gioria et al., 2012; Law et al., 2019). Despite these advantages, aquatic macrophytes remain insufficiently studied across large spatial scales (Alahuhta and Heino, 2013). We believe this research will help establish practical conservation priorities for freshwater habitats in Central Europe.

## Study sites

**Figure 1:**
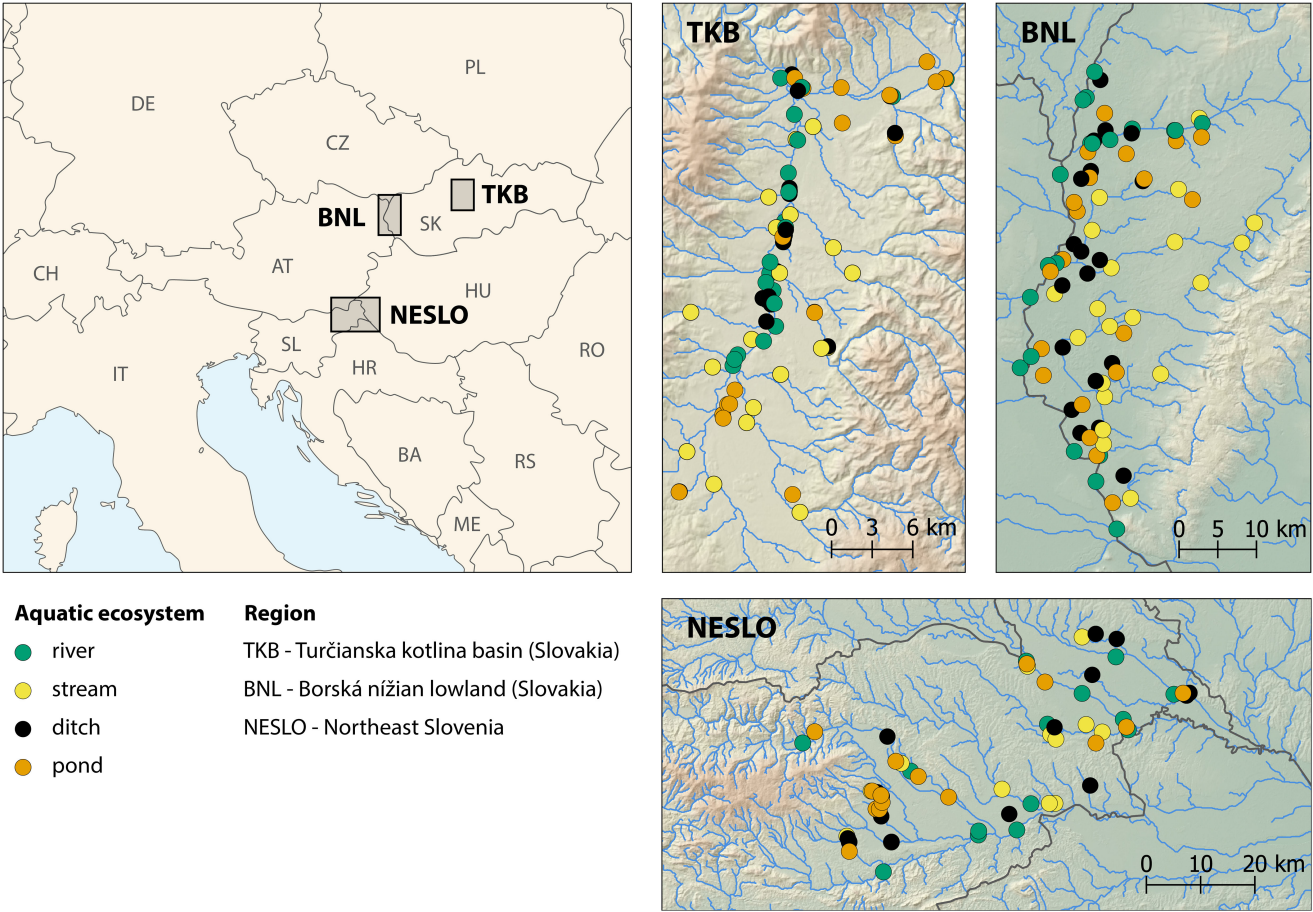
Location of the three studied regions in Central Europe and the positions of individual waterbodies within these regions.

The comparative diversity study was conducted in three model regions of Central Europe: the Turčianska kotlina Basin (TKB) in northern Slovakia (centered around 48.988°N, 18.883°E) within the Continental biogeographical region (see Cervellini et al., 2020; hereafter referred to as bioregion), the Borská nížina Lowland (BNL) in south-western Slovakia (~48.490°N, 17.072°E) within the Pannonian bioregion, and Northeast Slovenia (NESLO, ~46.566 N, 16.002 E), which lies at the transition between the Pannonian and Continental bioregions (**Figure 1**). These model areas mainly differ in altitude (mean altitude: TKB 437 m, min–max 383–576; BNL 161 m, 142–233; NESLO 226 m, 164–374) and associated climate. The mean annual temperature is lowest in TKB (7.4°C, 6.7–7.8), while the other two regions have the same temperature of about 9.8°C (BNL 9.3–10.1 and NESLO 9.2–10.3. Mean annual precipitation totals increase in the following order: BNL (695 mm, 641–823), TKB (805 mm, 794–838) and NESLO (944 mm, 772–1097). Additional details are given in **Supplementary Table S2**.

## Methods

### Sampling

Four types of aquatic ecosystems were explored within the studied regions: rivers, streams, ditches and ponds. We used classification criteria for waterbody types similar to those in previous studies on comparative diversity (Williams et al., 2004; Bubíková and Hrivnák, 2018b) (for details, see **Supplementary Table S1**). A total of 220 sites were randomly selected from all potential sites. Sampling sites were stratified by region (80 in Slovakia and 60 in Slovenia) and waterbody type (20 sites per waterbody type in Slovakia and 15 in Slovenia). Waterbodies were sampled for macrophytes during the early summer (June) and late summer (August and September) of 2011 in Slovakia and 2016 in Slovenia to minimize the effect of vegetation seasonality.

At each sampling site, an area of 100 m² was assessed, and the presence of all macrophytes (cf. Janauer and Dokulil, 2006) was recorded by walking or boat. The sampling area in rivers, streams and ditches was determined by the length × width of the watercourse. In ponds, the area was approximately triangular, with the apex at the centre of the waterbody and the base following the waterbody's margin. The smallest lentic waterbodies were excluded from potential sampling sites based on the specified sampling area (cf. Williams et al., 2004).

For the identification of red-list species, we used the lists published by Eliáš et al. (2015) for Slovakia and those presented in “https://www.uradni-list.si/files/RS_-2002-082-04055-OB~P001-0000.PDF” for Slovenia. Species nomenclature was standardized according to the Euro+Med PlantBase (https://europlusmed.org; for full names see **Supplementary Table S3**).

### Data analyses

Plant incidence data were summarized in species presence/absence matrices. We compared the diversity across the four waterbody types mentioned above within the three regions, examining three spatial scales: local (α) diversity, among-site (β) diversity, and regional (γ) diversity (Magurran, 2003). We conducted separate analyses on matrices for all recorded macrophyte species (total species), true aquatic plants (hydrophytes) and species of conservation concern (red-listed species).

#### Local (α) diversity

This study defined α diversity as the species richness within individual waterbodies. Differences in local diversity were assessed using generalized linear models (GLMs) with a Poisson error distribution and a logarithmic link function (McCullagh and Nelder, 1989). The GLMs included fixed effects for waterbody type (four levels: river, stream, ditch and pond), region (three levels: TKB, BNL and NESLO) and their interaction (waterbody × region). Model assumptions were examined using residual diagnostics. Due to considerable overdispersion, GLMs were refitted using a negative binomial distribution for overall α diversity and α diversity of hydrophytes (Hilbe, 2011). Statistically significant results from the overall GLM tests were followed by pairwise Tukey-type comparisons (Lenth, 2016).

#### Among-site (β) diversity

We defined β diversity as a variation in the species composition among particular sites. However, two different ecological phenomena can produce differences in species composition among sites: spatial turnover of species and nestedness of assemblages (Baselga, 2010, 2012). Species spatial turnover implies the replacement of species by others from site to site due to environmental sorting or spatial and historical constraints. In contrast, nestedness of species assemblages occurs when the species composition of poorer assemblages are nested subsets of richer assemblages as a consequence of various processes (e.g., Wright et al., 1997; Ulrich et al., 2009; Leprieur et al., 2011). Thus, we disentangled the turnover and nestedness component of β diversity following Baselga (2010, 2012). Specifically, we calculated Sørensen dissimilarity among sampling sites and additively partitioned this total dissimilarity measure (β_SOR_) to dissimilarity due to species replacement (a turnover component of dissimilarity: β_STU_) and dissimilarity due to nestedness (nestedness-resultant component of dissimilarity: β_SNE_). Differences in the total, turnover and nestedness-resultant components of β diversity among habitats within regions were assessed using distance-based tests for homogeneity of multivariate dispersion with 10,000 permutations (Anderson, 2006). Pairwise Tukey comparisons followed significant overall tests.

#### Regional (γ) diversity

We expressed γ diversity as the total number of species per habitat within each region. Randomization tests were employed to assess the null hypothesis that there are no differences in the total number of species among waterbody types within a given region. We used the differences in total species counts as the test statistic, comparing the observed values against a null distribution generated from 10,000 randomly reshuffled datasets (Manly, 2007). We calculated the probabilities of detecting differences greater than or equal to the observed value from these comparisons. Due to the unequal number of sites sampled in Slovakia (n = 20 per habitat) and Slovenia (n = 15), we employed sample-based rarefaction analysis to estimate the total number of taxa expected across fifteen sites per habitat and region. Ninety-five percent confidence intervals for each estimate were calculated using the analytical formulas provided by Colwell et al. (2004).

Analyses were performed in R (R Core Team, 2022), using the libraries *betapart* (Baselga et al., 2023), *ggplot2* (Wickham, 2016), *emmeans* (Lenth, 2023), *iNEXT* (Hsieh et al., 2022), *MASS* (Venables and Ripley, 2002) and *vegan* (Oksanen et al., 2022).

## Results

Altogether, 113 macrophyte taxa were identified across the study regions. Vascular plants comprised 86.1% of these taxa, followed by bryophytes – *Fontinalis antipyretica*, *Rhynchostegium riparioides* and *Riccia fluitans* – at 2.7% and macroscopic algae (identified to genus level as *Chara* and *Nitella*) at 1.8%. The highest number of macrophytes was detected in NESLO (94 taxa), followed by BNL (51) and TKB (43). All three regions had similarly high numbers of hydrophytes NESLO (24), THB (26) and BNL (22). The number of red-listed plants was comparable across the regions, with NESLO having 24, TKB 19 and BNL 17. The studied waterbodies were relatively species-poor, with macrophyte counts ranging from 0 to 22 in NESLO, 0 to 14 in BNL, and 0 to 10 in TKB. The most common plants in TKB were hydrophytes, with *Fontinalis antipyretica* at 30% and *Myriophyllum spicatum* at approximately 22%. In BNL, hydrophytes were also most frequent, with *Lemna minor* at around 42% and *Ceratophyllum demersum* at about 29%. In contrast, helophytes were most prevalent in NESLO, where *Phalaris arundinacea* reached 55% and *Agrostis stolonifera* approximately 37%. The most frequently found red-listed species were *Ranunculus aquatilis* in TKB (21%), *Potamogeton nodosus* in BNL (20%) and *Myriophyllum spicatum* and *Carex riparia* in NESLO (each at 25%).

Each waterbody type supported unique species that were not found in any other type, with the highest numbers in ponds and ditches and considerably fewer in streams and rivers. The number of species unique to ponds, ditches, rivers and streams was as follows: TKB – 13, 10, 3, 0; BNL – 7, 7, 2, 2; NESLO – 13, 11, 6, 6 (**Supplementary Table S4**). Ponds played an especially important role in supporting unique red-listed species. We recorded 4, 5 and 7 unique threatened species in TKB, BNL and NESLO ponds, respectively. Other aquatic ecosystems supported substantially fewer unique red-list species: in TKB, ditches had 2, rivers 3 and streams none; in BNL 3, 0 and 1; and in NESLO 2, 1 and 1 (for details see **Supplementary Table S4**).

### Local (α) diversity

**Figure 2:**
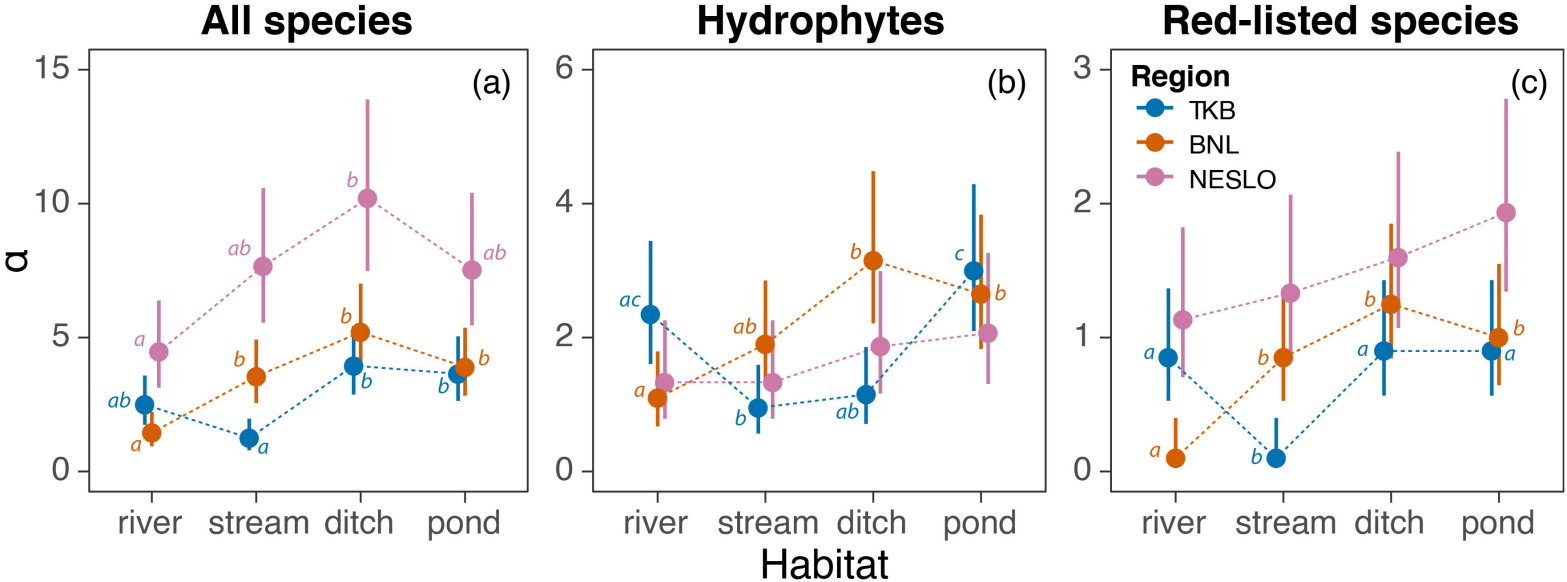
Comparison of macrophyte α diversity among aquatic ecosystems in the studied regions. Mean species richness values for all taxa **(a)**, hydrophyte species **(b)**, and red-listed species **(c)** are displayed (circles) along with 95% confidence intervals (error bars). Different lowercase letters indicate significant differences among habitat means within each region.

We did not find evidence for a general pattern of differences in α diversity among the aquatic ecosystems across the studied regions, as indicated by a significant interaction between region and habitat for all species (χ² = 19.7, df = 6, p = 0.003), hydrophytes (χ² = 21.2, df = 6, p = 0.002) and red-listed species (χ² = 30.5, df = 6, p < 0.001). Regarding all macrophyte species, ditches in NESLO supported significantly more species than rivers (**Figure 2a**). In TKB, rivers had significantly fewer species than the other waterbodies, while in BNL, streams had significantly fewer species than ditches and ponds.

For hydrophytes, no significant differences were observed in NESLO (**Figure 2b**). However, in TKB, ponds supported significantly more species than streams, while in BNL, ponds and ditches hosted more hydrophytes than rivers.

The diversity of red-listed species was comparable among the waterbodies in NESLO (**Figure 2c**). Nevertheless, rivers and streams harboured significantly fewer species of conservation concern than the other aquatic ecosystems in BNL and TKB, respectively.

### Among-site (β) diversity

**Figure 3:**
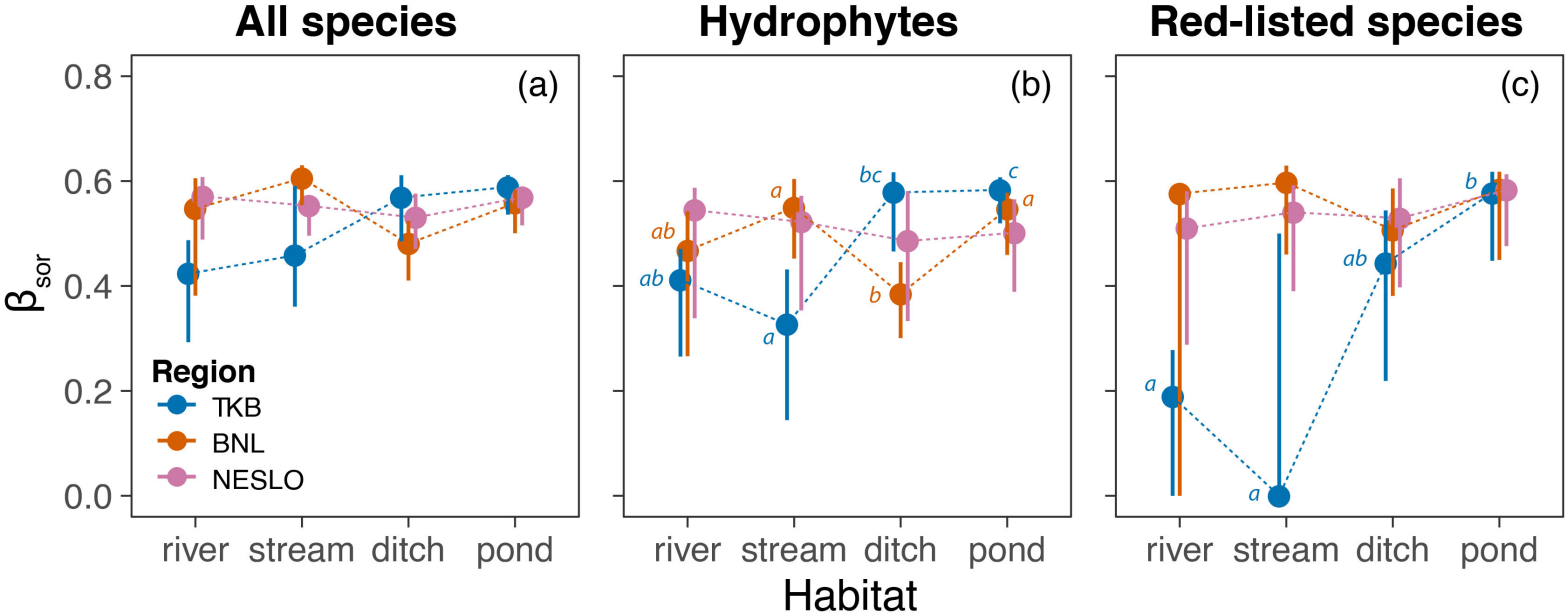
Comparison of macrophyte β diversity among aquatic ecosystems in the studied regions. Total β diversity (β_SOR_) of whole communities **(a)**, hydrophytes **(b)** and red-listed species **(c)** is displayed as distance to centroids in multivariate space (circles), along with 95% bootstrap confidence intervals (1,000 resamples). Different lowercase letters indicate significant differences among habitat means within each region. Results for the turnover and nestedness-resultant components of β diversity are given in **Supplementary Figure S1**.

For all macrophyte species, total β diversity was statistically comparable among habitats in NESLO (pseudo-F = 0.36, p = 0.78), BNL (pseudo-F = 2.15, p = 0.086), and TKB (pseudo-F = 2.08, p = 0.107) (**Figure 3a**). A more detailed examination of β diversity through additive partitioning revealed marginally significant differences in the turnover component in TKB (pseudo-F = 3.04, p = 0.031), where ditches and ponds showed greater heterogeneity due to species replacement compared to rivers (**Supplementary Figure S1a**). No significant differences were detected in the turnover components of β diversity for NESLO (pseudo-F = 0.50, p = 0.685) and BNL (pseudo-F = 2.73, p = 0.053), nor were there significant changes in the nestedness-related components of β diversity (NESLO: pseudo-F = 2.01, p = 0.119; BNL: pseudo-F = 2.01, p = 0.126; TKB: pseudo-F = 0.45, p = 0.731) (**Supplementary Figure S1b**).

We found significant differences in total β diversity among aquatic ecosystems in BNL (pseudo-F = 3.04, p = 0.033), TKB (pseudo-F = 4.78, p = 0.011) but not in NESLO (pseudo-F = 0.15, p = 0.926). In BNL, hydrophyte communities in ditches were significantly more homogeneous than those in ponds and streams (**Figure 3b**). In contrast, streams in TKB exhibited lower β diversity than ditches and ponds, while pond communities were more heterogeneous than rivers. This overall pattern of hydrophyte β diversity was driven by species turnover among waterbodies rather than by community nestedness (**Supplementary Figures S1c, d**).

The analysis of red-listed species revealed significant differences among aquatic ecosystems in TKB (pseudo-F = 5.07, p = 0.008) but not in BNL (pseudo-F = 0.57, p = 0.637) and NESLO (pseudo-F = 0.32, p = 0.818). Ponds in TKB showed significantly higher heterogeneity of red-listed species than rivers and streams (**Figure 3c**). Again, these differences in total β diversity were driven by species turnover among individual waterbodies, not by nestedness (**Supplementary Figures S1e, f**).

### Regional (γ) diversity

**Figure 4:**
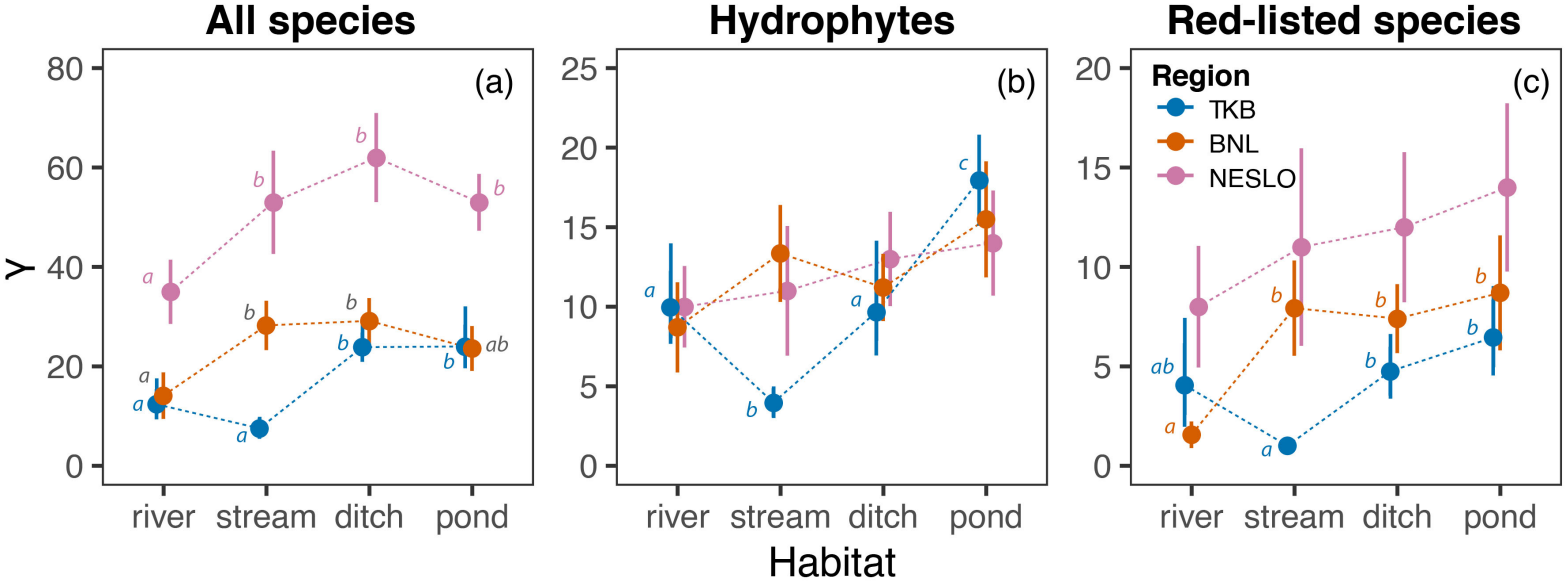
Comparison of aquatic macrophyte γ diversity among aquatic ecosystems in the studied regions. Overall γ diversity **(a)**, γ diversity of hydrophytes **(b)** and γ diversity of red-listed species **(c)** are estimated as the expected number of species in 15 sites. The estimates (circles) are shown along their 95% confidence intervals (error bars). Different lowercase letters indicate significant differences among habitat means within each region.

Ditches supported significantly more macrophyte species than rivers across all studied regions, with a similar trend observed in ponds, except in BNL (**Figure 4a**). Also, streams in NESLO and BNL harboured a high number of species, but their γ diversity was very low in TKB.

We did not find any significant differences in hydrophyte regional diversity among habitats in NESLO and BNL. In TKB, ponds supported significantly more species than the other habitats, while rivers and ditches hosted more hydrophytes than streams (**Figure 4b**).

Although the total number of red-listed species was similar across aquatic ecosystems in NESLO, ponds and ditches harboured more species of conservation concern than streams in TKB and rivers in BNL, respectively (**Figure 4c**). The relative importance of streams and rivers interchanged between TKB and BNL.

## Discussion

### Local (α) diversity

**Figure 5:**
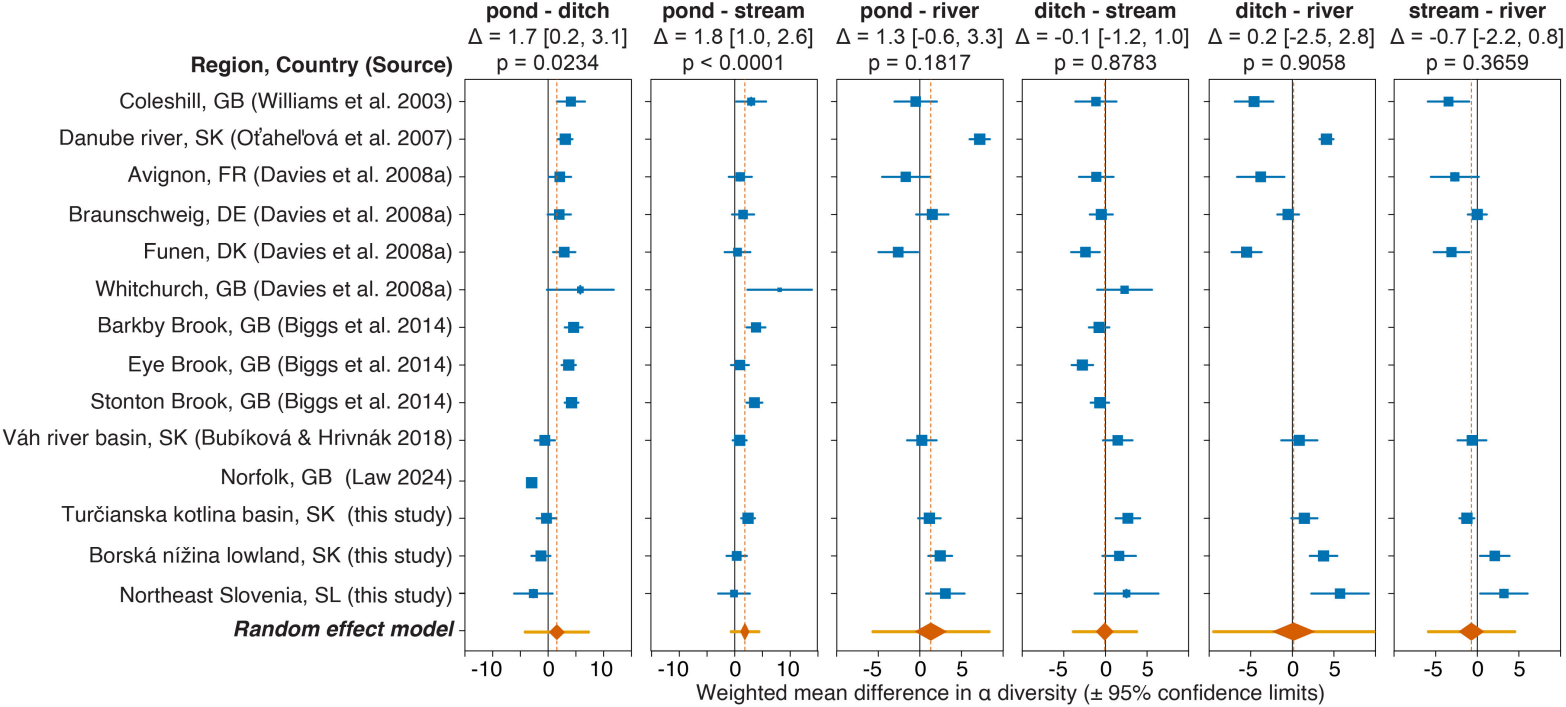
Forest plots showing differences in local (α) diversity of aquatic macrophytes among various habitats in Great Britain (GB), Denmark (DK), France (FR), Germany (DE), Slovenia (SL) and Slovakia (SK). Squares indicate the mean difference for each study, with their size proportional to the study's weight in the meta-analysis. Diamonds represent pooled estimates of mean differences based on random-effects models. Blue horizontal error bars and the sides of the diamonds denote 95% confidence intervals for individual studies and pooled estimates, respectively. Orange error bars indicate 95% prediction intervals for the random-effects models. Pooled estimates of mean differences (Δ) and their 95% confidence intervals (in square brackets) are displayed above each plot, along with the test results (p-values) for each meta-analytical model. Note that in Davies et al. (2008a), standard deviations were approximated from ranges using the normal distribution (two standard deviation rule, Higgins and Green, 2011).

Ponds and ditches exhibited higher α diversity than running waters across all regions, while the role of streams and rivers varied geographically. Ponds are generally known to support the high local diversity of macrophyte species (Williams et al., 2004; Biggs et al., 2005, 2007, 2017; Oťaheľová et al., 2007; Davies et al., 2008a, b; Fernández-Aláez et al., 2020). Based on these studies, local macrophyte diversity appears to decline in the following order: ponds (lakes) > rivers > streams > ditches. However, a comparative study of macrophyte richness across various waterbody types in Central Europe found that ditches, followed by rivers, exhibited species richness comparable to that of ponds (Bubíková and Hrivnák, 2018b). We conducted a meta-analysis using a random-effects model to synthesize findings from multiple comparative studies on macrophyte local diversity. The results indicate that ponds have significantly higher local diversity than both ditches and streams (**Figure 5**). No strong evidence was found for differences in local diversity among the other waterbody types. However, all comparisons exhibited considerable heterogeneity, as shown by significant Cochran’s Q-test results (all p-values < 0.01), elevated heterogeneity indices (all I^2^ values > 50%) and broad prediction intervals for effect sizes that included zero, suggesting a wide range of possible outcomes in future studies comparing the local diversity of these waterbodies. All studies confirmed higher diversity in ponds than in streams, but the comparison between ponds and ditches varied geographically. Ponds in Western Europe exhibited generally higher diversity than ditches (but see Law et al., 2024), while in Central and South-Eastern Europe, ditches had similar or even slightly higher diversity than ponds (see **Figure 2**; Bubíková and Hrivnák, 2018b). Our results and the meta-analysis highlight the role of ponds as macrophyte local diversity hotspots. High local diversity, along with a significant number of threatened species and species uniquely found in ponds – both in Central Europe and other regions (e.g., Linton and Goulder, 2000; Rhazi et al., 2012; Fois et al., 2024; Germ et al., 2024) – makes ponds habitats of high conservation priority within the European agricultural landscape.

We found that ditches supported the greatest number of macrophyte species recorded at individual sites in the studied regions. The high macrophyte diversity in ditches observed here and in Bubíková and Hrivnák (2018b) deviates markedly from the findings of many other comparative studies reporting lower macrophyte diversity in ditches and higher diversity in rivers (e.g., Williams et al., 2004; Biggs et al., 2007; Davies et al., 2008a). Moreover, the number of species unique to studied ditches was relatively high in all three regions. What explains the high diversity observed in artificial, man-made habitats such as ditches? In Central Europe, many ditches are historical structures built primarily in lowlands and basins during the intensification of agriculture and industrialization in the former Austro-Hungarian monarchy at the turn of the 19^th^ to 20^th^ century. These ditches were used for flood protection, agricultural drainage and/or as irrigation channels and are typical for relatively large size and permanent water levels (Dulovičová and Velísková, 2010). Due to political and economic changes in post-communist countries at the beginning of the 1990s, the current use of agricultural landscapes is much less intensive. Extensive soil fertilization and herbicide application were economically constrained, and many ditches were left to undergo the process of succession in areas of de-intensified or abandoned land (Bezák and Mitchley, 2014). As a result, these ditches have become species-rich habitats, supporting a relatively high proportion of endangered macrophyte species (Oťaheľová and Valachovič, 2002; Sipos et al., 2003; Dorotovičová, 2013). Other studies from European regions have shown that ditches can be diverse and provide exceptional conditions for aquatic plants (e.g., Armitage et al., 2003; Milsom et al., 2004; Biggs et al., 2007; Law et al., 2024). However, in several regions of Western Europe, ditches are often small, highly seasonal, located away from floodplain areas and close to intensively cultivated agricultural land. Using agrochemicals, such as herbicides, in these areas is likely to reduce macrophyte richness. Suboptimal hydrological conditions due to low water retention capacity and huge differences in run-off from cultivated areas, siltation and intensive agriculture may result in low biodiversity in these ditches (Williams et al., 2004; Davies et al., 2008a). Nevertheless, ditches have been shown to provide valuable wet, vegetated, non-cultivated habitats for both aquatic and terrestrial taxa, offer food resources and facilitate connectivity within the broader landscape (Herzon and Helenius, 2008). Despite their artificial origin, ditches play an important role in maintaining aquatic macrophyte diversity and supporting a large number of unique species in otherwise dry and intensively cultivated agricultural landscapes.

The comparison of local macrophyte diversity between and within regions revealed that diversity patterns are region-specific. The diversity trends in NESLO and BNL are similar, while they differ in TKB, particularly in streams. This pattern was observed across all studied species groups (**Figure 2**). Carpathian streams (TKB), in contrast to (sub)Pannonian streams (NESLO, BNL), retain a near-natural character but their macrophyte diversity is not equally high. Higher flow velocity, dominance of coarse sediments and heavy shading from riparian vegetation likely contribute to the naturally lower macrophyte species richness in these streams (Svitok et al., 2016). In general, only bryophytes and a few vascular aquatic plants are adapted to the conditions of European (sub-) mountain streams (Baattrup-Pedersen et al., 2006; Hrivnák et al., 2010).

### Among-site (β) diversity

Ponds consistently showed high β diversity, while the contribution of other aquatic ecosystems varied idiosyncratically across studied regions. In general, β diversity, or the differences in community composition among sites, increases either with dispersal limitations along spatial gradients or with species sorting along environmental gradients (Heino, 2011). Given the strong dispersal abilities of aquatic macrophytes (Santamaría, 2002) and considering that, despite several artificial barriers (Jones et al., 2020), the flat landscapes of the studied regions (max. altitudinal range of sites < 200 m) and relatively short distances between sites within regions, it is unlikely that contrasting β diversity patterns result from different dispersal processes between regions. Presumably, the contrasting patterns between regions are linked to habitat heterogeneity, often considered a key driver of β diversity (Suurkuukka et al., 2012; Astorga et al., 2014; Hamerlík et al., 2014). Most biotic communities are strongly influenced by environmental factors, and thus, habitat heterogeneity and the associated species sorting dynamics are thought to be the dominant mechanisms structuring communities (Cottenie, 2005). Based on the test of homogeneity of multivariate dispersion, the variability of habitat characteristics was comparable among waterbodies in NESLO (pseudo-F = 0.05, p = 0.99), while TKB and BNL exhibited significant heterogeneity (pseudo-F = 4.77, p = 0.007 and pseudo-F = 6.68, p = 0.002, respectively). These differences in habitat heterogeneity result in macrophyte communities with varying similarities, a pattern reflected in the differing β diversity among regions. Our findings suggest that β diversity patterns depend on region-specific environmental heterogeneity, precluding broader generalizations of the comparison.

In TKB, ponds exhibited a higher turnover component of β diversity than streams and rivers, regardless of whether the analysis included entire communities or focused on subsets such as hydrophytes and red-listed species. This pattern aligns with similar findings from other Western and Central European studies (Williams et al., 2004; Davies et al., 2008a; Bubíková and Hrivnák, 2018b). The consistency of these results suggests that common factors may sustain the high β diversity observed in ponds. The underlying mechanisms are likely related to habitat heterogeneity and connectivity, with plausible explanations of turnover species-sorting and patch-dynamics (Leibold et al., 2004; Fernández-Aláez et al., 2020). Ponds often have small catchment areas (Novikmec et al., 2016), resulting in highly variable physicochemical conditions that can differ significantly even across short distances (Svitok et al., 2011; Hamerlík et al., 2014). In contrast, rivers and streams typically have larger catchments, and the homogenizing effect of flowing water generally leads to more stable physical and chemical conditions than ponds. Analysis of physicochemical data from the TKB showed that ponds have significantly higher environmental heterogeneity than rivers (p = 0.004) and streams (p = 0.023). Sufficient dispersal within a heterogeneous environment and associated niche differences are expected to promote species sorting along resource gradients (Cottenie, 2005). As a result, this environmental heterogeneity may exert a niche-related influence on macrophyte communities, helping to sustain the high β diversity observed in ponds. Jeffries (2008) demonstrated that deterministic (i.e., niche-related) factors affecting macrophyte community variability are relevant even at very small spatial scales (much smaller than those investigated here), with the immediate surroundings of ponds playing a crucial role in shaping pond communities.

Spatial isolation may also contribute to the high β diversity of macrophytes in ponds. Unlike rivers and streams, which are highly connected water bodies where species dispersal is facilitated by fluvial action, ponds are more isolated, reducing species exchange and potentially enhancing β diversity. We observed a trend of increasing β diversity from fast-flowing to stagnant waters for all species and hydrophytes. However, this pattern was not evident for red-listed species, which were relatively scarce in the studied habitats (**Figure 2** and **Figure 3**). This is consistent with previous studies showing that high connectivity of lotic waterbodies may lead to more uniform vegetation and overall lower diversity of macrophytes (Tockner et al., 1998; Bornette et al., 1998, 2001). In contrast, small lentic habitats like ponds scattered within a hostile terrestrial matrix face a higher risk of local extinction and a lower likelihood of colonization (Wright et al., 1997). These stochastic processes are thought to contribute to the high heterogeneity observed in pond communities (Williams et al., 2004; Scheffer et al., 2006). However, empirical evidence directly linking isolation to increased variability in wetland plant communities remains limited. In fact, the effect of connectivity is one aspect of more complex metacommunity dynamics where habitat patches undergo both stochastic and deterministic extinctions that are counteracted by dispersal and where the environmental heterogeneity and inter-specific interactions shape species composition (Leibold et al., 2004; Scheffer et al., 2006). Even within strictly controlled experimental microcosms, aquatic plant communities did not display entirely deterministic behaviour, exhibiting a strong stochastic component in community assembly (Weiher and Keddy, 1995). We suggest that greater environmental heterogeneity and the small size and spatial isolation of ponds likely contribute to their elevated β diversity.

We demonstrated that macrophyte β diversity was predominantly driven by species turnover rather than nestedness. Our findings are consistent with global patterns in the β diversity of lake macrophytes, suggesting that natural environmental heterogeneity is the primary influence on macrophyte β diversity, with nestedness accounting for only a small fraction of the overall β diversity (Alahuhta et al., 2017). Nestedness, where communities with fewer species are subsets of richer communities, may arise due to factors like habitat size and isolation but these effects are often weak in freshwater systems (Heino, 2011). Given the strong dispersal abilities of macrophytes and the relatively small spatial scales explored in our study, it is unsurprising that species turnover is the dominant component of β diversity.

### Regional (γ) diversity

We have found that ponds and ditches harboured a high γ diversity of macrophytes, including red-listed species, across all studied regions, while the relative importance of ponds was especially pronounced in hydrophytes. The high regional diversity observed for ponds and ditches is likely related to their relatively high β diversity caused by higher heterogeneity of their environmental conditions and stochastic events (see above). Ponds, for example, often have small catchment areas (Novikmec et al., 2016), with great differences in land use, bedrock, management, and purpose resulting in significant gradients of physical and chemical conditions across the region (Svitok et al., 2011) that may promote greater biodiversity at the regional scale (Williams et al., 2004; Zelnik et al., 2012). Compared to other aquatic ecosystems, ponds have been shown to support the highest plant γ diversity across Europe (e.g., Williams et al., 2004; Biggs et al., 2005; Davies et al., 2008b; Lukács et al., 2013). In contrast, identifying ditches as the habitats with the highest regional diversity in Central Europe contradicts findings from Western Europe, where ditches differ in ecological characteristics, history and land use, leading to divergent results (Williams et al., 2004; Dorotovičová, 2013; Bubíková and Hrivnák, 2018b). Nevertheless, ponds exhibited the highest regional diversity of hydrophytes and red-listed plant species, highlighting their role as biodiversity hotspots with significant conservation value.

Regarding ponds, our results fully confirm the importance of these habitats for the maintenance of macrophyte diversity in Europe (Biggs et al., 2005, 2017). In contrast, ditches, which are considered relatively poor for plant species in Western Europe (Williams et al., 2004; Davies et al., 2008a), are among the richest in Central Europe for both wetland and Red List species (see also Bubíková and Hrivnák, 2018b).

## Conclusions

Ponds and ditches are critical habitats for biodiversity conservation within the European agricultural landscape. Despite their artificial origins, these small aquatic habitats support high local diversity, host a significant number of threatened and regionally unique species, and serve as vital refuges in predominantly dry and intensively cultivated areas. Ponds, in particular, stand out as biodiversity hotspots due to their high regional diversity of hydrophytes and red-listed plant species. Their small size, spatial isolation, and environmental heterogeneity likely contribute to their elevated β diversity, underscoring the importance of maintaining a network of these habitats to promote ecological connectivity and species persistence. Given their conservation value, creating, protecting and restoring ponds and ditches should be prioritized in agricultural land management strategies to preserve aquatic macrophyte biodiversity and the ecosystem services they provide.

## Data Availability

The raw data supporting the conclusions of this article will be made available by the authors, without undue reservation.
