# Supplementary figures and images for: Comparative diversity of aquatic plants in three Central European regions
Source: Front Plant Sci. 2025 Mar 6;16:1536731. doi: 10.3389/fpls.2025.1536731 (PMC11922903; doi:10.3389/fpls.2025.1536731)

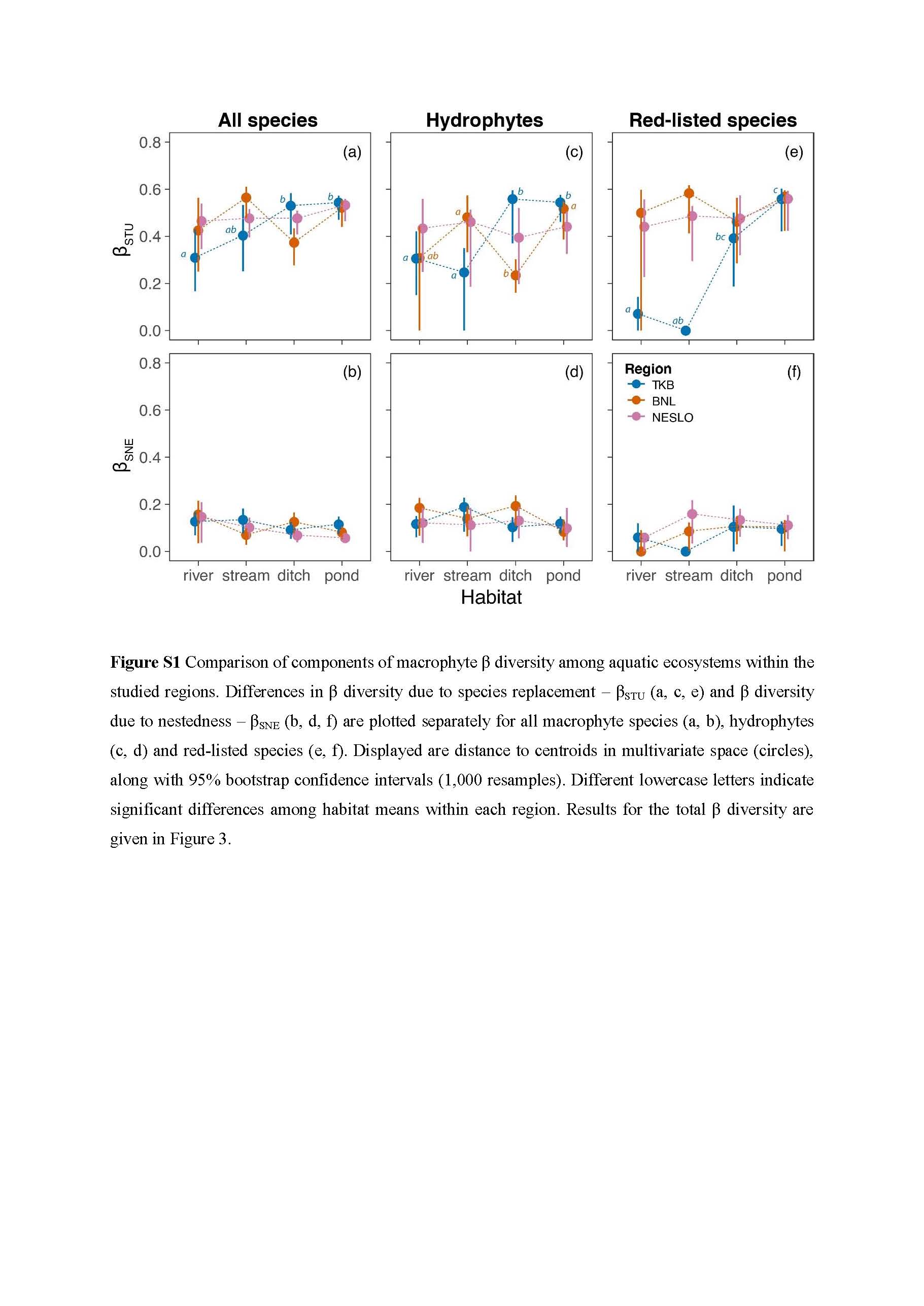

Supplement: Supplementary file 1 [file Image1.jpg]
