# Supplementary material for: Comparative diversity of aquatic plants in three Central European regions
Source: Front Plant Sci. 2025 Mar 6;16:1536731. doi: 10.3389/fpls.2025.1536731 (PMC11922903; doi:10.3389/fpls.2025.1536731)
Supplement: Supplementary file 2 [file Table1.docx]

**Table S1** Definitions of studied aquatic waterbody types.

| Waterbody | Definition |
| --- | --- |
| Rivers | Larger lotic waterbodies, created mainly by natural processes with a width of watercourses > 8 m |
| Streams | Small lotic waterbodies created mainly by natural processes with a width of watercourses < 8 m |
| Ponds | Lentic waterbodies which area is > 100 m^2^, including both man-made and natural waterbodies, which may be permanent or seasonal (reservoirs, gravel and sand pits, fishponds, river oxbows) |
| Ditches | Man-made canals created mainly for agricultural and industrial purposes and have i) a linear planform, ii) linear field boundaries, often turning at right angles, and c) little relationship with natural landscape contours |
