# Supplementary material for: Comparative diversity of aquatic plants in three Central European regions
Source: Front Plant Sci. 2025 Mar 6;16:1536731. doi: 10.3389/fpls.2025.1536731 (PMC11922903; doi:10.3389/fpls.2025.1536731)
Supplement: Supplementary file 3 [file Table2.docx]

**Table S2** Summary of environmental characteristics of studied regions.

|  | **NESLO** |  |  | **BNL** |  |  | **TKB** |  |  |
| --- | --- | --- | --- | --- | --- | --- | --- | --- | --- |
| **Environmental characteristics** | **Mean** | **Max** | **Min** | **Mean** | **Max** | **Min** | **Mean** | **Max** | **Min** |
| **Geographic characteristic** |  |  |  |  |  |  |  |  |  |
| ^1^Altitude (m) | 219.8 | 350.0 | 121.0 | 154.2 | 223.0 | 97.0 | 431.8 | 551.0 | 374.0 |
| **Vegetation characteristic** |  |  |  |  |  |  |  |  |  |
| ^2^Plant cover (%) | 31.1 | 100.0 | 0.0 | 34.1 | 100.0 | 0.0 | 46.1 | 100.0 | 0.0 |
| **^3^Landscape characteristics** |  |  |  |  |  |  |  |  |  |
| Tress & bushes (%) | 30.3 | 100.0 | 0.0 | 28.9 | 100.0 | 0.0 | 21.8 | 100.0 | 0.0 |
| Waters (%) | 5.9 | 85.0 | 0.0 | 10.2 | 99.5 | 0.0 | 5.1 | 70.0 | 0.0 |
| Grasslands (%) | 22.2 | 85.0 | 0.0 | 22.5 | 100.0 | 0.0 | 36.9 | 98.0 | 0.0 |
| Fields (%) | 33.7 | 100.0 | 0.0 | 29.3 | 100.0 | 0.0 | 17.6 | 95.0 | 0.0 |
| Urban and rural (%) | 8.1 | 60.0 | 0.0 | 9.5 | 95.0 | 0.0 | 16.1 | 90.0 | 0.0 |
| **^4^Substrate characteristics** |  |  |  |  |  |  |  |  |  |
| Fine (%) | 45.8 | 100.0 | 0.0 | 42.3 | 100.0 | 0.0 | 32.1 | 100.0 | 0.0 |
| Sand (%) | 12.8 | 72.7 | 0.0 | 25.0 | 100.0 | 0.0 | 8.9 | 100.0 | 0.0 |
| Gravel (%) | 21.8 | 100.0 | 0.0 | 12.5 | 100.0 | 0.0 | 26.1 | 100.0 | 0.0 |
| Stone and concrete (%) | 44.4 | 100.0 | 0.0 | 14.6 | 100.0 | 0.0 | 31.8 | 100.0 | 0.0 |
| **^5^Bank type** |  |  |  |  |  |  |  |  |  |
| Steep (%) | 97.0 | . | . | 73.7 | . | . | 66.2 | . | . |
| Gradual (%) | 3.0 | . | . | 26.3 | . | . | 33.8 | . | . |
| **Water characteristics** |  |  |  |  |  |  |  |  |  |
| ^6^Transparency |  |  |  |  |  |  |  |  |  |
| Clear | 58.0 | . | . | 2.5 | . | . | 31.3 |  |  |
| Transitional between clear and turbid | 17.0 | . | . | 40.0 | . | . | 45.0 |  |  |
| Turbid | 25.0 | . | . | 57.5 | . | . | 23.7 |  |  |
| ^7^pH | 7.0 | 8.8 | 5.8 | 7.9 | 9.4 | 6.1 | 8.0 | 8.7 | 6.9 |
| ^7^Conductivity (μS/cm) | 402.0 | 2150.0 | 62.0 | 580.3 | 1445.0 | 128.4 | 444.9 | 964.4 | 63.5 |
| ^8^Water depth (cm) | 80.6 | 630.0 | 5.6 | 90.5 | 285.0 | 7.1 | 62.7 | 300.0 | 7.5 |

Short methods: 1) Measured using Garmin equipment in the field; 2) Estimated in the field; 3) Estimated percentage of five landscape cover types within a 100 m radius around the sampling sites; 4) Estimated percentage of bottom substrate; 5) Bank type; 6) Turbidity based on three field observations in summer; 7) Measured in the field using CyberScan PC 650; 8) Average water depth from 10 randomly selected points at each sampling site.
