# Supplementary material for: Comparative diversity of aquatic plants in three Central European regions
Source: Front Plant Sci. 2025 Mar 6;16:1536731. doi: 10.3389/fpls.2025.1536731 (PMC11922903; doi:10.3389/fpls.2025.1536731)
Supplement: Supplementary file 4 [file Table3.docx]

**Table S3** List of scientific names of recorded vascular plant taxa in all studied regions.

*Agrostis stolonifera* L., *Alisma lanceolatum* With., *Alisma plantago-aquatica* L., *Alnus glutinosa* (L.) Gaertn., *Alopecurus geniculatus* L., *Berula erecta* (Huds.) Coville, *Bidens radiata* Thuill., *Bidens tripartita* L., *Butomus umbellatus* L., *Callitriche* L. .sp. div., *Caltha palustris* L., *Cardamine pratensis* L., *Carex acutiformis* Ehrh., *Carex bohemica* Schreb., *Carex brizoides* L., *Carex elata* All., *Carex hirta* L., *Carex pseudocyperus* L., *Carex riparia* Curtis, *Ceratophyllum demersum* L., *Ceratophyllum submersum* L., *Echinochloa crus-galli* (L.) P. Beauv., *Elodea canadensis* Michx., *Elode nuttallii* (Planch.) H. St. John, *Epilobium hirsutum* L., *Epilobium parviflorum* Schreb., *Epilobium* L. sp. div., *Epilobium tetragonum* L., *Equisetum arvense* L., *Equisetum palustre* L., *Ficaria verna* Huds., *Fontinalis antipyretica* Hedw., *Galium palustre* L., *Glechoma hederacea* L., *Glyceria maxima* (Hartm.) Holmb., *Glyceria fluitans* (L.) R. Br., *Glyceria notata* Chevall., *Helosciadium repens* (Jacq.) W. D. J. Koch, *Holcus lanatus* L., *Hottonia palustris* L., *Hydrocharis morsus-ranae* L., *Chara* L. sp. div., *Impatiens glandulifera* Royle, *Iris pseudacorus* L., *Juncus articulatus* L., *Juncus effusus* L., *Leersia oryzoides* (L.) Sw., *Lemna gibba* L., *Lemna minor* L., *Lemna trisulca* L., *Lycopus europaeus* L., *Lysimachia nummularia* L., *Lysimachia vulgaris* L., *Lythrum salicaria* L., *Mentha* ×*verticillata*, *Mentha aquatica* L., *Mentha longifolia* (L.) L., *Myosotis scorpioides* L., *Myriophyllum spicatum* L., *Myriophyllum verticillatum* L., *Najas marina* L., *Nasturtium officinale* W. T. Aiton, *Nitella* C.Agardh sp. div., *Nuphar lutea* (L.) Sm., *Nymphaea alba* L., *Oenanthe aquatica* (L.) Poir., *Persicaria amphibia* (L.) Delarbre, *Persicaria hydropiper* (L.) Delarbre, *Persicaria maculosa* Gray, *Persicaria dubia* (Stein) Fourr., *Phalaris arundinacea* (L.) Rauschert, *Phalaris arundinacea* cult., *Phragmites australis* (Cav.) Steud., *Portulaca oleracea* L., *Potamogeton berchtoldii* Fieber, *Potamogeton crispus* L., *Potamogeton lucesns* L., *Potamogeton natans* L., *Potamogeton nodosus* Poir., *Stuckenia pectinata* (L.) Börner, *Potamogeton perfoliatus* L., *Potamogeton pusillus* L., *Potentilla reptans* L., *Ranunculus aquatilis* agg., *Ranunculus circinans* (Julin) Ericsson, *Ranunculus repens* L., *Ranunculus trichophyllus* Chaix, *Rexnoutria japonica* agg., *Rhinchostegium riparioides* (Hedw.) Cardot, *Riccia fluitans* L., *Rorippa amphibia* (L.) Besser, *Rorippa palustris* (L.) Besser, *Rumex hydrolapathum* Huds, *Sagittaria sagittifolia* L., *Salix cinerea* L., *Salix euxina* I. V. Belyaeva, *Salix* L. sp. div., *Scirpus sylvaticus* L., *Scrophularia umbrosa* Dumort., *Scutellaria galericulata* L., *Schoenoplectus lacustris* (L.) Palla, *Solanum dulcamara* L., *Sparganium emersum* Rehmann, *Sparganium erectum* L., *Spirodela polyrhiza* (L.) Schleid., *Symphytum officinale* L., *Trapa natans* L., *Typha angustifolia* L., *Typha latifolia* L., *Utricularia vulgaris* agg., *Veronica anagalis-aquatica* L., *Veronica beccabunga* L., *Zannichellia palustris* L.
