# Supplementary material for: Comparative diversity of aquatic plants in three Central European regions
Source: Front Plant Sci. 2025 Mar 6;16:1536731. doi: 10.3389/fpls.2025.1536731 (PMC11922903; doi:10.3389/fpls.2025.1536731)
Supplement: Supplementary file 5 [file Table4.docx]

**Table S4** Unique species in waterbody types and individual studied region.

| \| **Region** \| **TKB** \|  \|  \|  \|  \| **BNL** \|  \|  \|  \|  \| **NESLO** \| \| \|  \| \| --- \| --- \| --- \| --- \| --- \| --- \| --- \| --- \| --- \| --- \| --- \| --- \| --- \| --- \| --- \| \| **Waterbody type** \| **ponds** \| **ditches** \| **rivers** \| **streams** \|  \| **ponds** \| **ditches** \| **rivers** \| **streams** \|  \| **ponds** \| **ditches** \| **rivers** \| **streams** \| \| **Plant taxa** \|  \|  \|  \|  \|  \|  \|  \|  \|  \|  \|  \|  \|  \|  \| \| *Agrostis stolonifera* \|  \| 2 \|  \|  \| *Agrostis stolonifera* \| 1 \|  \|  \|  \| *Alnus glutinosa* \|  \| 1 \|  \|  \| \| *Alisma plantago-aquatica* \| 1 \|  \|  \|  \| *Carex acutiformis* \|  \| 1 \|  \|  \| *Apium repens* \| 1 \|  \|  \|  \| \| *Berula erecta* \|  \| 7 \|  \|  \| *Elodea canadensis* \|  \|  \|  \| 1 \| *Batrachium aquatile* \|  \|  \| 1 \|  \| \| *Butomus umbellatus* \|  \|  \| 1 \|  \| *Fontinalis antipyretica* \|  \|  \| 2 \|  \| *Bidens radiata* \| 1 \|  \|  \|  \| \| *Callitriche* sp. \| 2 \|  \|  \|  \| *Glyceria fluitans* \|  \| 1 \|  \|  \| *Cardamine pratensis* \|  \| 1 \|  \|  \| \| *Ceratophyllum demersum* \| 3 \|  \|  \|  \| *Glyceria plicata* \|  \| 1 \|  \|  \| *Carex acutiformis* \|  \| 1 \|  \|  \| \| *Epilobium hirsutum* \|  \| 6 \|  \|  \| *Hottonia palustris* \| 1 \|  \|  \|  \| *Carex bohemica* \| 1 \|  \|  \|  \| \| *Glyceria plicata* \|  \| 2 \|  \|  \| *Lysimachia vulgaris* \|  \|  \| 1 \|  \| *Carex brizoides* \|  \|  \|  \| 1 \| \| *Juncus articulatus* \|  \| 1 \|  \|  \| *Nitella* sp. \|  \|  \|  \| 1 \| *Carex pseudocyperus* \| 2 \|  \|  \|  \| \| *Lemna minor* \| 6 \|  \|  \|  \| *Nymphaea alba* \| 1 \|  \|  \|  \| *Ceratophyllum demersum* \| 4 \|  \|  \|  \| \| *Lemna trisulca* \| 1 \|  \|  \|  \| *Persicaria hydropiper* \|  \| 1 \|  \|  \| *Ceratophyllum submersum* \| 2 \|  \|  \|  \| \| *Lysimachia nummularia* \| 1 \|  \|  \|  \| *Potamogeton lucesns* \| 1 \|  \|  \|  \| *Epilobium* sp*.* \|  \| 1 \|  \|  \| \| *Mentha aquatica* \|  \| 4 \|  \|  \| *Potamogeton perfoliatus* \|  \| 1 \|  \|  \| *Epilobium tetragonum* \|  \| 1 \|  \|  \| \| *Mentha longifolia* \|  \| 2 \|  \|  \| *Potamogeton berchtoldii* \|  \| 1 \|  \|  \| *Fallopia japonica* \|  \|  \| 1 \|  \| \| *Myosotis scorpioides* \|  \| 9 \|  \|  \| *Riccia fluitans* \| 1 \|  \|  \|  \| *Ficaria verna* \|  \|  \| 1 \|  \| \| *Myriophyllum verticillatum* \| 4 \|  \|  \|  \| *Scrophularia umbrosa* \|  \| 1 \|  \|  \| *Fontinalis antipyretica* \|  \|  \|  \| 1 \| \| *Najas marina* \|  \|  \| 1 \|  \| *Trapa natans* \| 2 \|  \|  \|  \| *Galium palustre* \|  \| 1 \|  \|  \| \| *Phalaroides arundinacea* \|  \| 1 \|  \|  \| *Typha angustifolia* \| 4 \|  \|  \|  \| *Holcus lanatus* \|  \|  \|  \| 1 \| \| *Phragmites australis* \| 1 \|  \|  \|  \|  \|  \|  \|  \|  \| *Hydrocharis morsus-ranae* \| 2 \|  \|  \|  \| \| *Potamogeton natans* \| 1 \|  \|  \|  \|  \|  \|  \|  \|  \| *Chara* sp. \|  \| 1 \|  \|  \| \| *Potamogeton pectinatus* \| 3 \|  \|  \|  \|  \|  \|  \|  \|  \| *Impatiens glandulifera* \|  \|  \|  \| 3 \| \| *Potamogeton perfoliatus* \|  \|  \| 1 \|  \|  \|  \|  \|  \|  \| *Lemna gibba* \|  \| 1 \|  \|  \| \| *Riccia fluitans* \| 1 \|  \|  \|  \|  \|  \|  \|  \|  \| *Mentha* ×*verticillata* \| 2 \|  \|  \|  \| \| *Scrophularia umbrosa* \|  \| 4 \|  \|  \|  \|  \|  \|  \|  \| *Mentha aquatica* \|  \| 1 \|  \|  \| \| *Typha latifolia* \| 5 \|  \|  \|  \|  \|  \|  \|  \|  \| *Myosotis scorpioides* \|  \|  \| 1 \|  \| \| *Utricularia vulgaris* agg. \| 4 \|  \|  \|  \|  \|  \|  \|  \|  \| *Myriophyllum verticillatum* \| 1 \|  \|  \|  \| \|  \|  \|  \|  \|  \|  \|  \|  \|  \|  \| *Najas marina* \| 1 \|  \|  \|  \| \|  \|  \|  \|  \|  \|  \|  \|  \|  \|  \| *Nasturtium officinale* \|  \|  \| 1 \|  \| \|  \|  \|  \|  \|  \|  \|  \|  \|  \|  \| *Phalaris arundinacea* cult. \| 1 \|  \|  \|  \| \|  \|  \|  \|  \|  \|  \|  \|  \|  \|  \| *Phellandrium aquaticum* \|  \|  \|  \| 1 \| \|  \|  \|  \|  \|  \|  \|  \|  \|  \|  \| *Portulaca oleracea* \|  \| 1 \|  \|  \| \|  \|  \|  \|  \|  \|  \|  \|  \|  \|  \| *Potamogeton perfoliatus* \|  \|  \| 1 \|  \| \|  \|  \|  \|  \|  \|  \|  \|  \|  \|  \| *Salix fragilis* \|  \|  \|  \| 1 \| \|  \|  \|  \|  \|  \|  \|  \|  \|  \|  \| *Salix* sp. \|  \| 1 \|  \|  \| \|  \|  \|  \|  \|  \|  \|  \|  \|  \|  \| *Symphytum officinale* \| 1 \|  \|  \|  \| \|  \|  \|  \|  \|  \|  \|  \|  \|  \|  \| *Utricularia vulgaris* agg. \| 1 \|  \|  \|  \| \| Total number of taxa \| 13 \| 10 \| 3 \| 0 \|  \| 7 \| 7 \| 2 \| 2 \|  \| 13 \| 11 \| 6 \| 6 \| \| Red-listed taxa \| 4 \| 2 \| 3 \| 0 \|  \| 5 \| 3 \| 0 \| 1 \|  \| 7 \| 2 \| 1 \| 1 \|   Legend: Plant taxa are presented with the number of occurrences in each waterbody type. Red List species are indicated by the red color. |  |  |  |  |  |  |  |  |  |  |  |  |  |  |
| --- | --- | --- | --- | --- | --- | --- | --- | --- | --- | --- | --- | --- | --- | --- | --- | --- | --- | --- | --- | --- | --- | --- | --- | --- | --- | --- | --- | --- | --- | --- | --- | --- | --- | --- | --- | --- | --- | --- | --- | --- | --- | --- | --- | --- | --- | --- | --- | --- | --- | --- | --- | --- | --- | --- | --- | --- | --- | --- | --- | --- | --- | --- | --- | --- | --- | --- | --- | --- | --- | --- | --- | --- | --- | --- | --- | --- | --- | --- | --- | --- | --- | --- | --- | --- | --- | --- | --- | --- | --- | --- | --- | --- | --- | --- | --- | --- | --- | --- | --- | --- | --- | --- | --- | --- | --- | --- | --- | --- | --- | --- | --- | --- | --- | --- | --- | --- | --- | --- | --- | --- | --- | --- | --- | --- | --- | --- | --- | --- | --- | --- | --- | --- | --- | --- | --- | --- | --- | --- | --- | --- | --- | --- | --- | --- | --- | --- | --- | --- | --- | --- | --- | --- | --- | --- | --- | --- | --- | --- | --- | --- | --- | --- | --- | --- | --- | --- | --- | --- | --- | --- | --- | --- | --- | --- | --- | --- | --- | --- | --- | --- | --- | --- | --- | --- | --- | --- | --- | --- | --- | --- | --- | --- | --- | --- | --- | --- | --- | --- | --- | --- | --- | --- | --- | --- | --- | --- | --- | --- | --- | --- | --- | --- | --- | --- | --- | --- | --- | --- | --- | --- | --- | --- | --- | --- | --- | --- | --- | --- | --- | --- | --- | --- | --- | --- | --- | --- | --- | --- | --- | --- | --- | --- | --- | --- | --- | --- | --- | --- | --- | --- | --- | --- | --- | --- | --- | --- | --- | --- | --- | --- | --- | --- | --- | --- | --- | --- | --- | --- | --- | --- | --- | --- | --- | --- | --- | --- | --- | --- | --- | --- | --- | --- | --- | --- | --- | --- | --- | --- | --- | --- | --- | --- | --- | --- | --- | --- | --- | --- | --- | --- | --- | --- | --- | --- | --- | --- | --- | --- | --- | --- | --- | --- | --- | --- | --- | --- | --- | --- | --- | --- | --- | --- | --- | --- | --- | --- | --- | --- | --- | --- | --- | --- | --- | --- | --- | --- | --- | --- | --- | --- | --- | --- | --- | --- | --- | --- | --- | --- | --- | --- | --- | --- | --- | --- | --- | --- | --- | --- | --- | --- | --- | --- | --- | --- | --- | --- | --- | --- | --- | --- | --- | --- | --- | --- | --- | --- | --- | --- | --- | --- | --- | --- | --- | --- | --- | --- | --- | --- | --- | --- | --- | --- | --- | --- | --- | --- | --- | --- | --- | --- | --- | --- | --- | --- | --- | --- | --- | --- | --- | --- | --- | --- | --- | --- | --- | --- | --- | --- | --- | --- | --- | --- | --- | --- | --- | --- | --- | --- | --- | --- | --- | --- | --- | --- | --- | --- | --- | --- | --- | --- | --- | --- | --- | --- | --- | --- | --- | --- | --- | --- | --- | --- | --- | --- | --- | --- | --- | --- | --- | --- | --- | --- | --- | --- | --- | --- | --- | --- | --- | --- | --- | --- | --- | --- | --- | --- | --- | --- | --- | --- | --- | --- | --- | --- | --- | --- | --- | --- | --- | --- | --- | --- | --- | --- | --- | --- | --- | --- | --- | --- | --- | --- | --- | --- | --- | --- | --- | --- | --- | --- | --- | --- | --- | --- | --- | --- | --- | --- | --- | --- | --- | --- | --- | --- | --- | --- | --- | --- | --- | --- | --- | --- | --- | --- | --- | --- | --- | --- | --- | --- | --- | --- | --- | --- | --- | --- | --- | --- | --- | --- | --- | --- | --- | --- | --- | --- | --- | --- | --- | --- | --- | --- | --- | --- | --- | --- | --- | --- | --- | --- | --- | --- | --- | --- | --- | --- | --- | --- | --- | --- | --- | --- | --- | --- | --- | --- | --- | --- | --- | --- | --- | --- | --- | --- | --- | --- | --- | --- | --- | --- | --- | --- | --- | --- | --- | --- | --- | --- | --- | --- | --- | --- | --- | --- | --- | --- | --- | --- | --- | --- | --- | --- | --- | --- | --- | --- | --- | --- | --- |
